# Unilateral optic nerve infiltration as an initial site of relapse of acute lymphoblastic leukemia in remission

**DOI:** 10.4103/0974-620X.71902

**Published:** 2010

**Authors:** Sabyasachi Bandyopadhyay, Debabrata Das, Gobinda Das, Sibnath Gayen

**Affiliations:** Department of Ophthalmology and Pediatrics, R.G. Kar Medical College and Hospital, 1, Khudiram Bose Sarani, Kolkata - 700 004, India

Acute lymphoblastic leukemia (ALL) is the most common childhood cancer and 70–80% are cured with modern chemotherapy.[1] Reports of central nervous system (CNS) infiltration in relapsing leukemia are becoming more nowadays because of increased survival rate following recent treatment.[2,3] Active bone marrow disease is usually associated with optic nerve infiltration.[4] Isolated optic nerve involvement is rarely reported as an initial site of relapse after complete systemic remission.[5,6] Here we describe a nine-year-old patient who was diagnosed to have ALL at the age of six years and received conventional chemotherapy with complete remission. He developed a right sided optic nerve relapse three years later without systemic involvement.

**Figure 1:**
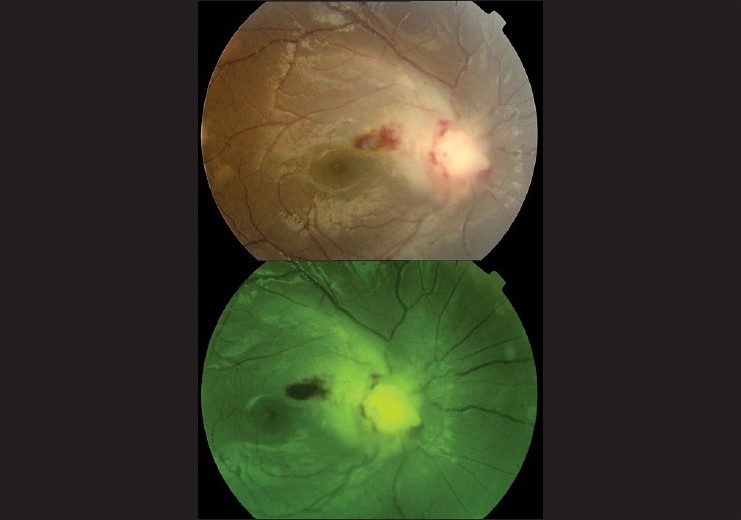
Fundus photography of right eye showing optic disc edema and retinal hemorrhage

**Figure 2:**
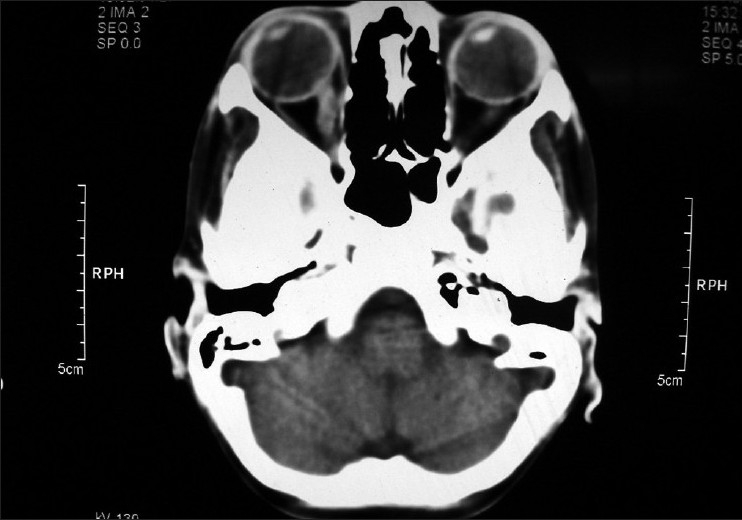
Axial computed tomography (CT) scan of brain and orbit showing thickened right optic nerve

**Figure 3:**
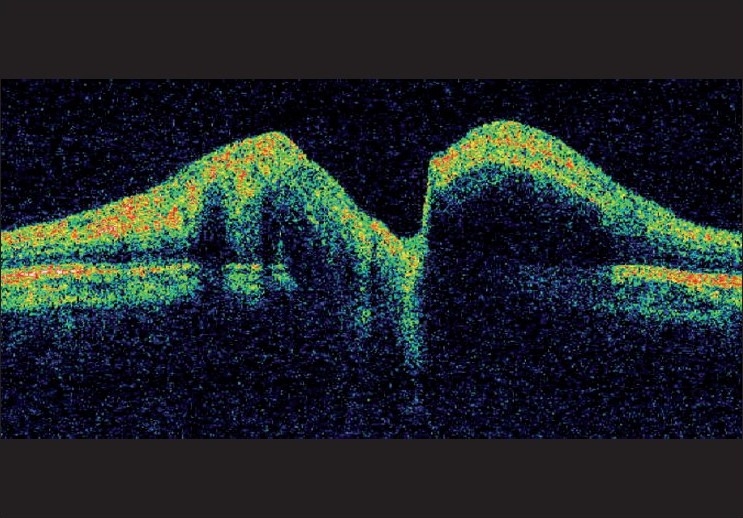
The optical coherence tomography (OCT) of optic nerve head (ONH) showing swollen right optic nerve head

In April 2006, a six-year-old boy presented with intermittent high grade fever, marked weight loss, generalized lymphadenopathy, splenomegaly and gum bleeding. The complete blood count (CBC) revealed a hemoglobin level of 7.2 g/dl, leukocyte count of 23 × 10^9^/l with blast cells in smear and platelet count of 110 × 10^9^/l. Bone marrow examination was suggestive of pre-B cell acute lymphoblastic leukemia (ALL). Standard risk induction chemotherapy comprising vincristine 1.4 mg/m^2^, daunorubicine 60 mg/m^2^, prednesolone 70 mg/m^2^ and L-asparaginase 10,000 U/m^2^ was instituted. Complete remission was achieved in seven weeks. Then intrathecal methotrexate, cytosine arabinoside and hydrocortisone were given to the child every two months and intravenous vincristine started in October 2006 for two and a half years. Cerebrospinal fluid (CSF) examination was done prior to every intrathecal drug instillation and thereafter showed no evidence of CNS disease. In March 2009, the child complained of dimness of vision in his right eye. His best corrected visual acuity (BCVA) in right eye was 20/200 and in left eye was 20/20. Color vision was abnormal in right eye. On ophthalmoscopy, right sided optic disc edema and peripapillary nerve fiber layer edema were detected [Figure 1]. There was peripapillary retinal hemorrhage but vitreous, macula and retinal vessels were normal. Left eye was normal. A relative afferent pupillary defect (RAPD) was also noticed in the right side. The computed tomography (CT) scan of brain and orbit showed thickened right optic nerve [Figure 2]. The optical coherence tomography (OCT) of optic nerve head (ONH) indicated swollen right ONH [Figure 3]. A CSF study revealed few leukemic cells. CBC and bone marrow examination was normal. Then intrathecal methotrexate was administered along with cranial irradiation with teletheraphy by Co 60 (total dose 20 Gy in 10 fractions spread over two weeks). He was followed up for six months. His BCVA in right eye improved to 20/80 only probably due to radiation optic neuropathy. His color vision also improved with reduction of optic disc edema and subsidence of retinal hemorrhage.

The optic nerve is relatively unaffected by systemic chemotherapy and serves as a sanctuary of ALL.[7] So, for early diagnosis of ALL relapse in addition to periodic CBC, bone marrow examination, CSF study, a regular ophthalmic check up, CT and MRI should be done following complete remission. To confirm the diagnosis of optic nerve infiltration, other causes of decreased visual acuity in ALL during remission like infection, vasculitis, radiation optic neuropathy or side effects of chemotherapeutic agents have to be excluded. To the best of our knowledge, thorough MEDLINE search, optic nerve infiltration in ALL relapse has been rarely reported in literature.[5,6]

Therefore, optic nerve as an initial site of recurrence of ALL should always be kept in mind as prompt treatment can improve visual outcome and long-term survival rate.
